# Supplementary material for: Characterization of genetic and molecular tools for studying the endogenous expression of Lactate dehydrogenase in Drosophila melanogaster
Source: PLoS One. 2024 Jan 3;19(1):e0287865. doi: 10.1371/journal.pone.0287865 (PMC10763966; doi:10.1371/journal.pone.0287865)
Supplement: S1 Fig — The Ldh-mCherryGenomic spatial expression pattern is consistent with previous studies, with Ldh-mCherryGenomic being expressed at high levels in the body wall muscle. However, unlike Ldh-GFPGenomic, the expression of mCherryGenomic fusion protein persists throughout much of pupal development (compare with Fig 1B). (PDF) [file pone.0287865.s001.pdf]

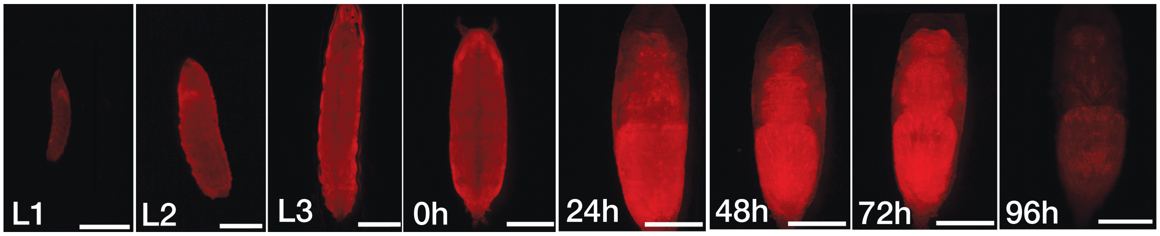

Figure S1. Expression of *Ldh-mCherry*^{Genomic} during larval development. The *Ldh-mCherry*^{Genomic} spatial expression pattern is consistent with previous studies, with Ldh-mCherry^{Genomic} being expressed at high levels in the body wall muscle. However, unlike Ldh-GFP^{Genomic}, the expression of mCherry^{Genomic} fusion protein persists throughout much of pupal development (compare with Figure 1B).
