# Supplementary material for: Characterization of genetic and molecular tools for studying the endogenous expression of Lactate dehydrogenase in Drosophila melanogaster
Source: PLoS One. 2024 Jan 3;19(1):e0287865. doi: 10.1371/journal.pone.0287865 (PMC10763966; doi:10.1371/journal.pone.0287865)
Supplement: Attachment — Submitted filename: Response to Reviewers Comments_v2.docx [file pone.0287865.s005.docx]

Response to Reviewers Comments:

Reviewer #1: All comments were adequately addressed. There are a few very minor changes I recommend before final publication:
*1) I agree that Abu-Shumays et al. is sufficient evidence for the claim that Ldh expression is relatively high during larval developement. But, it seems this citation was added to the sentence prior to this claim (Line 79). I recommend moving it to the sentence discussion Ldh expression (Line 80).*

Done.

*2) The method for combining Z-stacks is still not provided (e.g. maximum intensity projection / average intensity projection). I recommend this is added to the sentence on Line 196.*

We appreciate the suggestion, but would kindly correct the reviewer and note that the methods sections is accurate and does not need edits. As already stated in the text, we did not combine Z-stacks in the figure, but rather used a representative section.

*3) Figure 4 and 5 have the green channel legend labeled αLDH whereas figure 2 and everywhere else in the body text and figure legends denotes this without all uppercase as αLdh. I recommend changing figure 4 and 5 to match everywhere else (i.e. αLdh).*

Done.

*Reviewer #2: I consider that the authors have responded adequately to all my comments. I recommend the publication of this study.*

Thank you!
